# Antimicrobial susceptibility testing results from 13 hospitals in Viet Nam: VINARES 2016–2017

**DOI:** 10.1186/s13756-021-00937-4

**Published:** 2021-05-10

**Authors:** Tien Viet Dung Vu, Marc Choisy, Thi Thuy Nga Do, Van Minh Hoang Nguyen, James I. Campbell, Thi Hoi Le, Vu Trung Nguyen, Heiman F. L. Wertheim, Ngoc Thach Pham, Van Kinh Nguyen, H. Rogier van Doorn, the VINARES consortium

**Affiliations:** 1 Oxford University Clinical Research Unit, National Hospital for Tropical Diseases, 78 Giai Phong, Dong Da, Hanoi, Viet Nam; 2 National Hospital for Tropical Diseases, Hanoi, Viet Nam; 3 Centre for Tropical Medicine and Global Health, Nuffield Department of Medicine, University of Oxford, Oxford, UK; 4 Department of Medical Microbiology, Radboudumc Center for Infectious Diseases, Radboudumc, Nijmegen, Netherlands

**Keywords:** Antimicrobial resistance, Surveillance, Viet Nam, VINARES

## Abstract

**Objective:**

To analyse data from 2016–17 from a hospital-based antimicrobial resistance surveillance with national coverage in a network of hospitals Viet Nam.

**Methods:**

We analysed data from 13 hospitals, 3 less than the dataset from the 2012–13 period. Identification and antimicrobial susceptibility testing data from the clinical microbiology laboratories from samples sent in for routine diagnostics were used. Clinical and Laboratory Standards Institute 2018 guidelines were used for antimicrobial susceptibility testing interpretation. WHONET was used for data entry, management and analysis.

**Results:**

42,553 deduplicated isolates were included in this analysis; including 30,222 (71%) Gram-negative and 12,331 (29%) Gram-positive bacteria. 8,793 (21%) were from ICUs and 7,439 (18%) isolates were from invasive infections. *Escherichia coli* and *Staphylococcus aureus* were the most frequently detected species with 9,092 (21%) and 4,833 isolates (11%), respectively; followed by *Klebsiella pneumoniae* (3,858 isolates – 9.1%) and *Acinetobacter baumannii* (3,870 isolates – 9%). Bacteria were mainly isolated from sputum (8,798 isolates – 21%), blood (7,118 isolates – 17%) and urine (5,202 isolates – 12%). Among Gram-positives 3,302/4,515 isolates (73%) of *S. aureus* were MRSA; 99/290 (34%) of *Enterococcus faecium* were resistant to vancomycin; and 58% (663/1,136) of *Streptococcus pneumoniae* proportion were reduced susceptible to penicillin. Among Gram-negatives 59% (4,085/6,953) and 40% (1,186/2,958) of *E. coli* and *K. pneumoniae* produced ESBL and 29% (376/1,298) and 11% (961/8,830) were resistant to carbapenems, respectively. 79% (2855/3622) and 45% (1,514/3,376) of *Acinetobacter spp.* and *Pseudomonas aeruginosa* were carbapenem resistant, respectively. 88% (804/911) of *Haemophilus influenzae* were ampicillin resistant and 18/253 (7%) of *Salmonella spp.* and 7/46 (15%) of *Shigella spp.* were resistant to fluoroquinolones. The number of isolates from which data were submitted in the 2016–2017 period was twice as high as in 2012–2013. AMR proportions were higher in 2016–2017 for most pathogen-antimicrobial combinations of interest including imipenem-resistant *A. baumannii*, *P. aeruginosa* and Enterobacterales*.*

**Conclusions:**

The data show alarmingly high and increasing resistant proportions among important organisms in Viet Nam. AMR proportions varied across hospital types and should be interpreted with caution because existing sampling bias and missing information on whether isolates were community or hospital acquired. Affordable and scalable ways to adopt a sample- or case-based approach across the network should be explored and clinical data should be integrated to help provide more accurate inferences of the surveillance data.

**Supplementary Information:**

The online version contains supplementary material available at 10.1186/s13756-021-00937-4.

## Introduction

In a 2015 estimate based on data from the European Antimicrobial Resistance Surveillance Network (EARS-Net), over 33,000 (out of 445 million inhabitants) people die each year in the European Union as a direct consequence of drug resistant infections [1]. Data from low- and middle-income countries (LMICs) are rare, but a recent paper from Thailand – with a population of 69 million – estimated that 19,122 of 45,209 (43%) deaths in patients with hospital-acquired infections are due to drug resistant infections. This higher number of deaths per capita attributable to AMR in Thailand in comparison with the EU suggests the burden of AMR in LMICs may be higher [2].

In their 2014 review, Rossolini et al*.* indicated an out-of-control crisis for Gram-negative pathogens, particularly with the worrisome emergence and spread of carbapenem-resistant Enterobacterales, especially in the hospital environment, while Gram-positive pathogens appear to be relatively under control [3].

In May 2015, the World Health Assembly adopted a Global Action Plan on Antimicrobial Resistance, which highlighted the need to improve awareness and understanding of antimicrobial resistance and to strengthen the knowledge and evidence-based decisions through surveillance and research [4]. The review by the World Health Organisation (WHO) pointed out the lack of a global consensus on methodology and data collection for AMR surveillance. In addition, routine surveillance often uses samples from severe cases including those with hospital acquired infections and those with treatment failure, leading to an under-representation of samples from patients with community-acquired infections (CAI) and failure of the data to properly inform treatment guidelines [5]. As a response to this situation, WHO introduced that same year the Global Antimicrobial Resistance Surveillance System (GLASS). GLASS aims to enable standardized, comparable and validated AMR data collection and analysis and sharing of AMR data across countries to inform decision-making and action [6].

AMR surveillance activities were initiated in Viet Nam in 1988 with specific programs as summarised previously [7], including VINARES, a network of 16 hospitals throughout the country collecting data on antimicrobial consumption and resistance and hospital-acquired infections [7]-[10].

These projects highlighted the high proportions of resistance among several WHO GLASS target pathogens: carbapenem-resistant *Acinetobacter baumannii* (40% in the Global Antibiotic Resistance Partnership (GARP) in 2009 [11] and 70% in VINARES in 2012 [7]); *Escherichia coli* and *Klebsiella pneumoniae* producing extended spectrum beta-lactamase (ESBL) (30% and 43% in 2009, respectively); carbapenem-resistant *E. coli* (2% in 2009 [11] and 6% in 2012 [7]); carbapenem-resistant *K. pneumoniae* (10% in 2009 [11] and 17% in 2012 [7]); methicillin-resistant *Staphylococcus aureus* (MRSA), reported at 30.1% among hospital-acquired infections in 2004 [12] and at 69% among all isolates 2012 [7].

In 2013, the Viet Nam Ministry of Health published its national action plan on AMR, including strengthening and improving the national surveillance system on the use of antimicrobials and drug resistance [13]. In 2015, Viet Nam received pilot funding from the Fleming Fund to establish a National AMR surveillance network and reference laboratory [10]. The VINARES network was recognised in 2016 by the Ministry of Health as the national AMR surveillance network and continues to receive support from the Fleming Fund as part of the country grant for Viet Nam led by FHI360. The national AMR surveillance network also receives support from the US Centers for Disease Control and Prevention (US CDC) and Program for Appropriate Technology in Health (PATH) as part of the Global Health Security Agenda. A surveillance protocol based on GLASS and the Fleming Fund roadmap is being developed by the Ministry of Health with support from US CDC, WHO and Oxford University Clinical Research Unit (OUCRU). Data collection as part of a project on development on evidence based guidelines restarted in 2016 [10].

Here, we present the identification and antimicrobial susceptibility testing (AST) results from isolates from clinical specimens from 13 microbiology laboratories participating in VINARES between June 2016 and May 2017. These results provide an insight in the dynamics of AMR and an update on the earlier results published based on data from the VINARES for the 2012–2013 period [7].

## Materials and methods

### Data collection

**Fig. 1:**
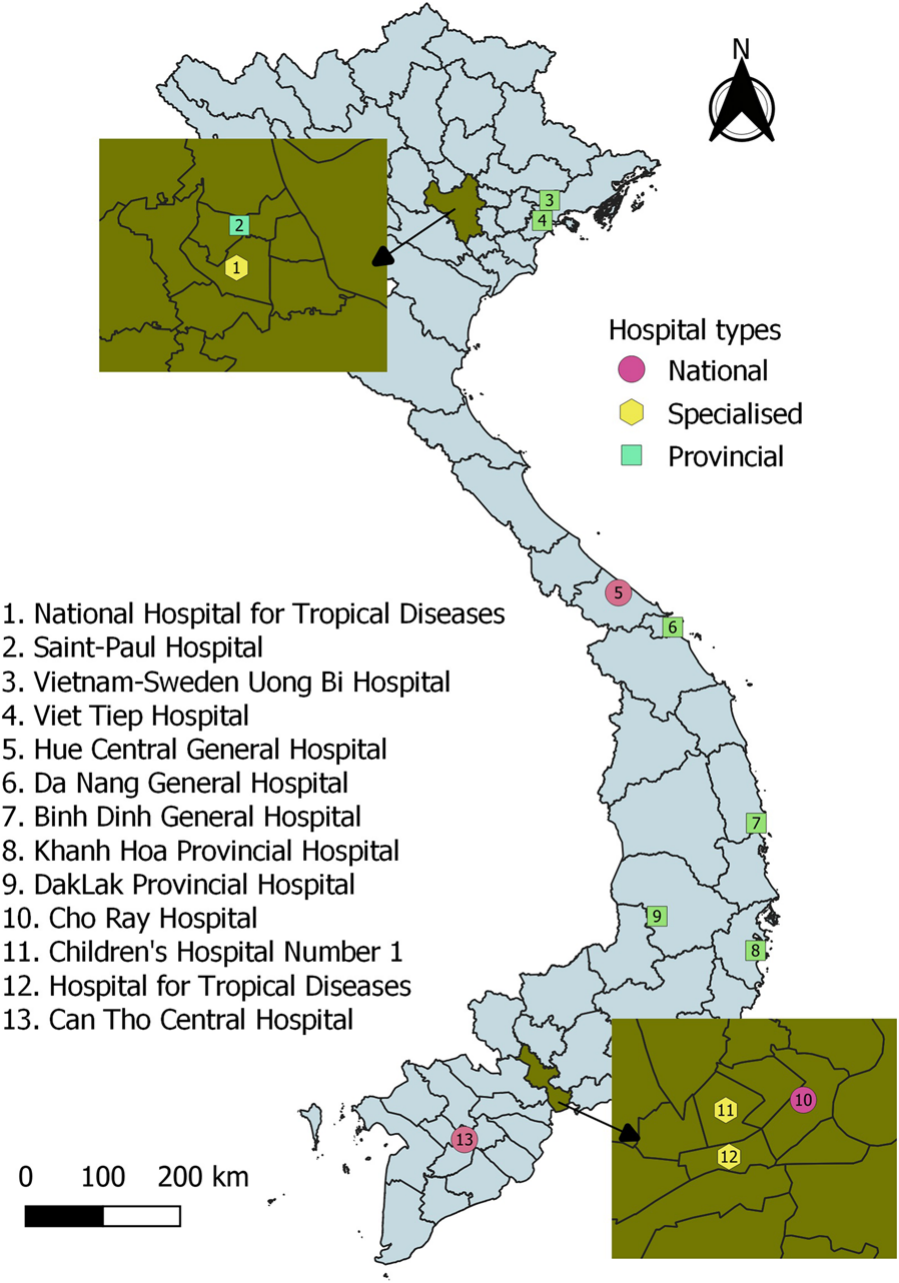
Fig. 1 Location, speciality, and type of the 13 participating hospitals in the VINARES 2016–2017 project

The VINARES network was described previously [7, 8]. In 2016–2017, 13 hospitals (7 provincial, 3 specialised and 3 national) continued to participate in the network, among which 4 were in the northern, 5 in the central and 4 in the southern region of Viet Nam; there were 1 paediatric and 2 infectious diseases hospitals (Fig. 1).

Thirteen hospitals submitted their routine identification and AST results quarterly by email from May 2016 to April 2017. These were results from all bacterial isolates from clinical specimens sent in for routine diagnostics at the microbiology lab of the hospitals. WHONET was used for data entry, management and analysis [14]. Routine AST data at the participating laboratories was entered into WHONET 5.6 by hospital technicians or was exported from automated systems including VITEK2 (bioMérieux, Marcy l’Etoile, France) or Phoenix automated microbiology system (BD Diagnostic Systems, Sparks, MD, USA) using LABCONN (LabSoft, Viet Nam). Raw data files were extracted and submitted by email. Files were converted to WHONET format using BacLink, a free tool included in WHONET [15]. All data files were combined into a single file. Data files were checked for common errors and compatibility (language and file structure). Sites had received regular training from OUCRU and National Hospital for Tropical Diseases staff since 2012, were enrolled into an external quality assurance scheme (UK-NEQAS) and received Vietnamese translated Clinical and Laboratory Standards Institute (CLSI) guidelines.

AST results were obtained by disk diffusion (DD) and minimum inhibitory concentration (MIC) methods. The proportion of MIC testing depended on the laboratory, the specific pathogen-antimicrobial combination and the period of study as detailed in Additional file 1:  table S1. AST results were categorised according to the CLSI 2018 guidelines as susceptible, intermediate, resistant or unknown. For each pathogen and antimicrobial under surveillance, the proportion of patients with growth of resistant bacteria was calculated in all specimens, and separately in specimens from Intensive Care Units (ICU), invasive infection (blood and cerebrospinal fluid (CSF)) or stool (for *Shigella spp.* and *Salmonella spp.*). AST results were interpreted using WHONET (version 5.6) and then summarized in R software [16].

MRSA was assessed using oxacillin and cefoxitin screening. As not all hospitals used molecular or other confirmation testing, an *S. aureus* isolate was considered MRSA if it was resistant to one of these two antimicrobials. In 2012–13, reduced susceptibility to penicillin in *Streptococcus pneumoniae* was mostly detected using oxacillin screening [7]. In 2016–17 this was more commonly done directly by penicillin susceptibility testing using both disk diffusion and MIC by E-test or automated systems. Oxacillin susceptibility results were used in case of missing penicillin susceptibility testing results.

We included five antibiotic classes: carbapenems (imipenem, meropenem and ertapenem), aminoglycosides (amikacin, gentamicin and tobramycin), fluoroquinolones (ciprofloxacine and levofloxacine), macrolides (azithromycin, erythromycin and clindamycin) and cephalosporins (ceftriaxone and cefepime).

Multidrug Resistant (MDR) and Extensively drug resistant (XDR) *E. coli*, *K. pneumoniae*, *Pseudomonas aeruginosa*, *A. baumannii* and *S. aureus* were defined as follows:

- *E. coli* and *K. pneumoniae* MDR: non-CRE and resistant to one third-generation cephalosporin, ciprofloxacin and one aminoglycoside.
- *E. coli* and *K. pneumoniae* XDR: carbapenem resistant and resistant to one third-generation cephalosporin, ciprofloxacin and one aminoglycoside.
- *P. aeruginosa* MDR: resistant to three of the following four antibiotics: imipenem, ceftazidime, ciprofloxacin and tobramycin [17].
- *A. baumannii* MDR: resistant to at least one agent in three of the following four antibiotic classes: carbapenems, aminoglycosides, fluoroquinolones and cephalosporins [18].
- *S. aureus* MDR: MRSA.

### Statistical analysis

We analysed data for eleven pathogens: *A. baumannii*, *P. aeruginosa*, *E. coli*, *K. pneumoniae*, *Enterobacter spp.*, *Enterococcus faecium*, *S. aureus*, *S. pneumoniae*, *Haemophilus influenzae, Salmonella spp.* and *Shigella spp.* [19]*.* Data were de-duplicated, so that one isolate represents one patient. Only the first isolate per patient, per pathogen, per reporting period, per stratification level (hospital) was included. This also minimized bias associated with reporting of repeat cultures [20]. Local specimen types were converted into specimen types compatible with WHONET.

An analysis of antibiotic resistance by hospital type was carried out. Three hospital types were considered: national and provincial level general and specialised hospitals, as shown in Fig. 1. Among the 16 hospitals participating in VINARES 2012–2013, three (one national and two specialised, all in the northern region) did not participate in 2016–2017 period. Data from each hospital type were pooled and analysed. This analysis served to compare susceptibility between hospital types. Only the pathogen-antimicrobial combinations with the highest sample numbers were selected, including imipenem-resistant *A. baumannii*, *E. coli*, *ESBL*-producing *E. coli* and *MRSA*.

Resistant proportions of pathogen-antimicrobial combinations between two periods of VINARES were compared using Chi-square test (significance level = 0.05).

## Results

### Distribution of bacteria and antibiotics

Between May 2016 and April 2017, hospitals submitted results from 75,051 specimens. Among them, 22,752 records were unknown or reported no growth, 48,084 were from Gram-negative and Gram-positive bacteria, 882 were fungi, 1454 were anaerobes, 1,864 were mycobacteria and 15 were parasites.

After removal of negative cultures, fungi, anaerobes, mycobacteria and parasites and deduplication, results from 42,553 isolates were included in the analysis; including from 30,222 (71%) Gram-negative and 12,331 (29%) Gram-positive bacteria. Among all isolates, 8,793 (21%) were from ICUs and 7,439 (18%) were from invasive infections.

*E. coli* and *S. aureus* were the most frequently isolated species with 9,092 (21%) and 4,833 isolates (11%), respectively; followed by *K. pneumoniae* (3,870 isolates – 9%) and *A. baumannii* (3,710 isolates – 9%). Bacteria were mainly isolated from sputum (8,798 isolates – 21%), blood (7,118 isolates – 17%) and urine (5,202 isolates – 12%); 321 isolates (1%) were from cerebrospinal fluid (CSF).

AST results were obtained by disk diffusion (DD) and MIC methods. Details by laboratory, period, and bacteria-drug combination are described in Table S1. Two laboratories used 100% DD in the first period and 100% MIC in the second period, one laboratory used MIC in the first period and DD in second period, and others kept 100% DD for MRSA screening in both periods. Among the 13 hospitals participating in the two periods, the number of hospitals that used MIC for ESBL testing increased from 6 to 12. As a result, we observed an increase in the number of ESBL-producing *E. coli* and K. *pneumoniae* tests (1659 in the first period and 9911 in the second period). This increase might also be because more hospitals switched from manual to automated systems, and ESBL were tested for all samples and not just to confirm third-generation cephalosporins resistance. Two laboratories in the 2^nd^ period of VINARES used MIC for imipenem-resistance testing versus only one in the 1^st^ period. Penicillin-susceptibility of *S. pneumoniae* were tested using MIC by three laboratories with 86/344 (25%) tests in the 1^st^ period, while they were tested using MIC in six laboratories with 694/1,136 (61%) tests in the 2^nd^ period.

### Antibiotic susceptibility testing results of Gram-positive bacteria

Antimicrobial susceptibility testing results of bacteria from all specimens and from invasive infections or stool are shown in Tables 1, 2, 3, 4, respectively. Additional file 1: Table 2a and 2b shows AST results from ICUs.

**Table 1:** Antimicrobial susceptibility testing results of three gram-positive bacteria in all specimens of 13 hospitals in VINARES 2016–2017 project. Denominators and numerators are the numbers of tested resistant isolates respectively. Corresponding resistant percentages are in brackets

	*S. aureus* (N = 4833)	*S. pneumoniae* (N = 1367)	*E. faecium* (N = 296)
Aminoglycosides	1674/4090 (41%)		34/46 (74%)
Fluoroquinolones	1720/4618 (37%)	31/1117 (3%)	
Macrolides	3861/4661 (83%)	1234/1317 (94%)	249/262 (95%)
Penicillin	2347/2400 (98%)	663/1136 (58%)	111/124 (90%)
SXT	1021/4158 (25%)	886/1069 (83%)	73/77 (95%)
Ampicillin	57/64 (89%)	2/21 (10%)	228/253 (90%)
Vancomycin	45/2680 (2%)*	16/1229 (1%)	91/290 (31%)

SXT: Trimethoprim/Sulfamethoxazole

**Table 2:** Antimicrobial susceptibility testing results of eight gram-negative bacteria in all specimens of 13 hospitals in VINARES 2016–2017 project. Denominators and numerators are the numbers of tested and resistant isolates respectively. Corresponding resistant percentages are in brackets

	*E. coli* (N = 9092)	*K. pneumoniae* (N = 3870)	*A. baumannii* (N = 3710)	*P. aeruginosa* (N = 3461)	*Enterobacter spp.* (N = 1322)	*H. influenzae* (N = 1085)	*Salmonella spp.* (N = 277)	*Shigella spp.* (N = 53)
Carbapenem	961/8830 (11%)	1049/3816 (27%)	2855/3622 (79%)	1514/3376 (45%)	376/1298 (29%)	0/1065 (0%)	1/195 (1%)	1/19 (5%)
Aminoglycosides	4188/8785 (48%)	1756/3780 (46%)	2686/3641 (74%)	1457/3389 (43%)	637/1297 (49%)		48/78 (62%)	4/5 (80%)
Fluoroquinolones	5813/8682 (67%)	1593/3619 (44%)	2929/3589 (82%)	1435/3357 (43%)	484/1271 (38%)	7/909 (1%)	18/253 (7%)	7/46 (15%)
Cephalosporins	5441/8195 (66%)	1995/3732 (53%)	2969/3549 (84%)	1392/3058 (46%)	675/1192 (57%)	18/664 (3%)	20/217 (9%)	8/26 (31%)
Macrolides			25/29 (86%)			4/1015 (0%)	53/137 (39%)	2/3 (67%)
SXT	5704/7843 (73%)	1753/3348 (52%)		1329/1388 (96%)	467/929 (50%)	429/470 (91%)	39/237 (16%)	44/50 (88%)
AMC	1476/3251 (45%)	1080/1999 (54%)			461/604 (76%)	271/358 (76%)		
Ampicillin	5547/5938 (93%)	2563/2622 (98%)			476/510 (93%)	804/911 (88%)	104/252 (41%)	35/46 (76%)
TCC	1317/2947 (45%)	863/1449 (60%)		1097/2160 (51%)	297/671 (44%)			

SXT: Trimethoprim/Sulfamethoxazole; AMC: amoxicillin clavulanic acid; TCC: Ticarcillin/Clavulanic Acid; *: Resistant and Intermediate

**Table 3:** Antimicrobial susceptibility testing results in blood and CSF of three gram-positive bacteria of 13 hospitals in VINARES 2016–2017 project. Denominators and numerators are the numbers of tested and resistant isolates respectively. Corresponding resistant percentages are in brackets

	*S. aureus* (N = 715)	*S. pneumoniae* (N = 160)	*E. faecium* (N = 51)
Aminoglycosides	294/637 (46%)		7/9 (78%)
Fluoroquinolones	297/689 (43%)	2/143 (1%)	
Macrolides	545/693 (79%)	140/152 (92%)	46/48 (96%)
Penicillin	490/504 (97%)	42/114 (37%)	19/22 (86%)
SXT	233/661 (35%)	107/134 (80%)	20/20 (100%)
Ampicillin			37/40 (92%)
Vancomycin	7/565 (1%)^*^	4/148 (3%)	13/51 (25%)

SXT: Trimethoprim/Sulfamethoxazole

**Table 4:** Antimicrobial susceptibility testing results in blood and CSF of *A. baumannii*, *P. aeruginosa*, *E. coli*, *K. pneumoniae*, *Enterobacter spp.*, *E. faecium*, *S. aureus*, *S. pneumoniae* and *H. influenzae*; in stool for *Salmonella spp.* and *Shigella spp.* of 13 hospitals in VINARES 2016–2017 project. Denominators and numerators are the numbers of tested and resistant isolates respectively. Corresponding resistant percentages are in brackets

	*E. coli* (N = 1535)	*K. pneumoniae* (N = 482)	*A. baumannii* (N = 187)	*P. aeruginosa* (N = 142)	*Enterobacter spp.* (N = 77)	*Shigella spp.* (N = 37)**	*Salmonella spp.* (N = 32)**	*H. influenzae* (N = 12)
Carbapenem	116/1483 (8%)	109/476 (23%)	110/183 (60%)	54/139 (39%)	20/77 (26%)	1/14 (7%)	0/19 (0%)	0/11 (0%)
Aminoglycosides	637/1471 (43%)	195/470 (41%)	107/185 (58%)	48/138 (35%)	35/75 (47%)			
Fluoroquinolones	953/1475 (65%)	177/459 (39%)	96/182 (53%)	37/138 (27%)	24/76 (32%)	4/31 (13%)	3/27 (11%)	0/9 (0%)
Cephalosporins	931/1402 (66%)	221/471 (47%)	118/178 (66%)	47/120 (39%)	37/66 (56%)	7/21 (33%)	4/28 (14%)	0/11 (0%)
Macrolides			1/1 (100%)			2/3 (67%)	1/5 (20%)	0/4 (0%)
SXT	935/1377 (68%)	215/454 (47%)		74/82 (90%)	29/57 (51%)	31/34 (91%)	6/30 (20%)	5/8 (62%)
AMC	180/577 (31%)	112/285 (39%)			26/32 (81%)			1/5 (20%)
Ampicillin	928/1028 (90%)	278/287 (97%)			21/23 (91%)	23/33 (70%)	14/31 (45%)	7/8 (88%)
TCC	169/356 (47%)	115/195 (59%)		46/110 (42%)	14/45 (31%)			

SXT: Trimethoprim/Sulfamethoxazole; AMC: amoxicillin clavulanic acid; TCC: Ticarcillin/Clavulanic Acid; *: Resistant and Intermediate

Since not all isolates were tested for all listed antibiotics, the denominator of each susceptible proportion test was different and smaller than the total number of isolates collected. There were 4,833 *S. aureus* isolates, including 715 (15%) from blood and CSF. 690 isolates (14%) were from ICU. 73% (3,302/4,515 isolates) of *S. aureus* were MRSA, 71% of *S. aureus* (476/674) from blood and CSF were MRSA. Among the isolates from ICU, the proportion of MRSA was 75% (478/640). The proportion reported as non-susceptible to vancomycin was low (2% (45/2,680) in all specimens and 1% (7/565) in blood and CSF). No confirmatory testing for vancomycin resistance was reported. The proportion resistant to macrolides was 83% (38,61/4,661) in all specimens.

*E. faecium* was isolated from 296 specimens; among which 51 (17%) were blood and CSF and 65 (22%) were from ICU. 34/46 tested isolates (74%) were high level aminoglycoside-resistant, 7/9 isolated from blood and CSF. 99/290 isolates (34%) of *E. faecium* were resistant to vancomycin (VRE) (19% of VRE tests were done by MIC method). 22 of 64 isolates (36%) from ICU were reported as vancomycin-resistant.

1,367 *S. pneumoniae* were isolated among which 160 (12%) were from blood and CSF and 184 isolates (13%) were from ICU. The penicillin-resistant *S. pneumoniae* proportion was 58% (663/1,136) in all specimens, and lower in blood and CSF (37%, 42/114 isolates) and among isolates from specimens collected in ICU (29%, 42/146 isolates). 691/794 (87%) of penicillin susceptibility tests were done by MIC method. 58/356 (16%) *S. pneumoniae* isolates were cephalosporin-resistant; this proportion was lower among ICU isolates (11%, 10/94). Two isolates (0.2%) were recorded as resistant to vancomycin, none of them were from blood/CSF or ICU.

### Antibiotic susceptibility testing results of Gram-negative bacteria

The numbers of *K. pneumoniae, E. coli* and *Enterobacter spp.* were 3,870, 9,092 and 1,322, respectively. In blood and CSF, these proportions were 12% (482/3,870), 17% (1,535/9,092) and 6% (77/1,322) in same order. The proportions of *K. pneumoniae*, *E. coli* and *Enterobacter spp.* isolated from ICUs were 28% (1,069/3,870), 11% (1,016/9,092 isolates) and 17% (230/1,322), respectively. The proportion of *E. coli* carrying ESBL was 59% (4,085/6,953) and 40% (1,186/2,958) in *K. pneumoniae*. Carbapenem-resistance among *K. pneumoniae*, *E. coli* and *Enterobacter spp.* was 29% (376/1,298), 11% (961/8,830) and 27% (1,049/3,816), respectively. Trimethoprim/sulfamethoxazole-resistance ranged from 47% (215/454) of *K. pneumoniae* in blood and CSF to 76% (700/925) of *E. coli* in ICU. MDR proportions of *E. coli* and *K. pneumoniae* were 29% (2,015/6,956) and 14% (428/3,141), respectively. There were 514/6,956 (7%) of *E. coli* and 722/3,141 (23%) of *K. pneumoniae* classified as XDR.

The number of isolates of *A. baumannii* and *P. aeruginosa* were similar (3,710 and 3,461, respectively). 187 (5%) isolates of *A. baumannii* and 482 (13%) of *P. aeruginosa* were isolated from blood and CSF. A high proportion of *A. baumannii* and *P. aeruginosa* isolates were from ICU (32% (1,176/3,710) and 33% (1,158/3,461), respectively). Ceftazidime-resistant proportions of *A. baumannii* in all specimens and in ICU were 2,743/3,298 (83%) and 866/958 (90%). These resistant proportions in *P. aeruginosa* were 1,378/3,231 (43%) and 574/1,062 (54%). Carbapenem-resistant proportions of *A. baumannii* and *P. aeruginosa* were 79% (2,855/3,622) and 45% (1,514/3,376), respectively. Out of 1,566 *P. aeruginosa* tested with the four selected antibiotics, 660 isolates (42%) were MDR. 2,781/3,442 (81%) of the tested *A. baumannii* isolates were MDR.

Of 1,085 *H. influenzae* isolates submitted, 146 were from ICU and 12 were from blood and CSF. The proportion of ampicillin-resistant *H. influenzae* was 88% (804/911) among all isolates; this proportion was higher among isolates collected on ICU (92/98 isolates – 94%). Three percent (18/664) of *H. influenzae* isolates were cephalosporins-resistant, while none were found resistant to carbapenems.

*Salmonella spp.* and *Shigella spp.* susceptibility were investigated in all specimens and in stool. Among 277 isolates of *Salmonella spp.*, there were 32 isolates from stool and 18 isolates from ICU. Fluoroquinolones-resistant *Salmonella spp.* in all specimens and in stool were 7% (18/253 and 11% (3/27), respectively. Among 53 *Shigella spp.* isolates, 70% came from stool. 7/46 (15%) of *Shigella spp.* were fluoroquinolones-resistant.

### Susceptibility by hospital type

Carbapenem-resistant *A. baumannii*, ESBL positive *E. coli* and MRSA in national, provincial general and specialised hospitals were compared, as the number of these pathogen-antimicrobial combinations were high enough for reliable comparison. Details are shown in Additional file 1: Table 3. *A. baumannii* had the highest carbapenem resistant proportion in national level hospitals, followed by specialised and provincial level hospitals (82% (1,979/2,413), 77% (444/577) and 68% (432/632), respectively). *E. coli* showed a different ESBL positive proportion between national and provincial level hospitals (58% (2,145/3,726) and 65% (1,541/2,371), respectively). MRSA proportions were lower in provincial (71% (1,499/2,115)) and specialised hospitals (72% (688/960)) than in national level hospitals (77% (1,115/1,440)).

### Comparison with data from VINARES 2012–2013

We compared the susceptibility of bacteria-antimicrobial combinations between the two periods of VINARES (2012–2013 versus 2016–2017). Laboratories used similar protocols in the two periods, including antimicrobial susceptibility testing methods using translated CLSI guidelines and data collection procedures. Laboratories were enrolled in the UK-NEQAS external quality assessment programme during both data collection periods. Since the VINARES 2016–2017 had 13 hospitals, we calculated the antimicrobial susceptibility result of VINARES 2012–2013 in whole dataset and in a subset of 13 hospitals. Table 5 shows resistant proportions of priority pathogen-antimicrobial combinations between the two periods.

**Table 5:** Resistance proportion of priority bacteria-antimicrobial combinations in all specimens and in blood and CSF, in 2012 and 2016. Denominators and numerators are the numbers of tested and resistant isolates respectively. Corresponding resistant percentages are in brackets

	Bacteria	All specimens			Blood and CSF (stool for *Salmonella spp.* and *Shigella spp.*)		
		2012 (16 hospitals)	2012 (13 hospitals)	2016	2012 (16 hospitals)	2012 (13 hospitals)	2016
ESBL	*E. coli*	1337/1928 (69%)	626/844 (74%)	4085/6953 (59%)	126/183 (69%)	59/81 (73%)	655/1107 (59%)
Imipenem	*E. coli*	180/2 977 (6%)	145/2111 (7%)	687/8438 (8%)	15/403 (4%)	9/309 (3%)	92/1410 (7%)
Ceftriaxone	*E. coli*	2342/4 192 (56%)	776/1472 (53%)	5051/7049 (72%)	240/514 (47%)	114/234 (49%)	912/1324 (69%)
MDR	*E. coli*	453/1828 (25%)	441/1639 (27%)	2015/6956 (29%)	24/125 (19%)	24/125 (19%)	336/1204 (28%)
XDR	*E. coli*	71/1828 (4%)	63/1639 (4%)	514/6956 (7%)	2/125 (2%)	2/125 (2%)	65/1204 (5%)
ESBL	*K. pneumoniae*	887/1400 (63%)	555/815 (68%)	1186/2958 (40%)	91/172 (53%)	34/61 (56%)	128/365 (35%)
Imipenem	*K. pneumoniae*	393/2 294 (17%)	259/1697 (15%)	891/3647 (24%)	64/361 (18%)	26/233 (11%)	91/454 (20%)
Ceftriaxone	*K. pneumoniae*	1479/2 227 (66%)	626/1380 (45%)	1912/3436 (56%)	101/190 (53%)	63/175 (36%)	214/435 (49%)
MDR	*K. pneumoniae*	318/1553 (20%)	294/1315 (22%)	428/3141 (14%)	17/112 (15%)	17/112 (15%)	53/403 (13%)
XDR	*K. pneumoniae*	205/1553 (13%)	171/1315 (13%)	722/3141 (23%)	12/112 (11%)	12/112 (11%)	81/403 (20%)
Imipenem	*A. baumannii*	1495/2138 (70%)	1056/1584 (67%)	2769/3551 (78%)	110/244 (45%)	85/205 (41%)	100/178 (56%)
MDR	*A. baumannii*	897/1334 (67%)	897/1282 (70%)	2781/3442 (81%)	27/44 (61%)	27/44 (61%)	101/171 (59%)
Imipenem	*P. aeruginosa*	578/1 765 (33%)	322/996 (32%)	1403/3220 (44%)	36/129 (28%)	22/88 (25%)	49/135 (36%)
MDR	*P. aeruginosa*	178/576 (31%)	144/392 (37%)	660/1566 (42%)	4/25 (16%)	4/17 (24%)	17/70 (24%)
MRSA	*S. aureus*	1 098/1 580 (69%)	950/1303 (73%)	3302/4515 (73%)	145/197 (74%)	130/171 (76%)	476/674 (71%)
Vancomycin^*^	*S. aureus*	28/823 (3.4%)	10/372 (2%)	45/2680 (2%)	5/135 (3.7%)	0/65 (0%)	7/565 (1%)
Penicillin	*S. pneumoniae*	115/344 (33%)^**^	115/341 (34%)^**^	663/1136(58%)	7/30 (23%) ^**^	7/30 (23%) ^**^	42/114 (37%)
Ceftriaxone	*S. pneumoniae*	90/358 (25%)	31/299 (10.4%)	57/352 (16%)	9/52 (17%)	4/47 (8.5%)	17/125 (14%)
Vancomycin	*E. faecium*	20/79 (25%)	20/79 (25%)	91/290 (31%)	2/14 (14%)	2/14 (14%)	13/51 (25%)
Ampicillin	*H. influenzae*	160/226 (71%)	1/1 (100%)	804/911 (88%)	3/5 (60%)	1/1 (100%)	7/8 (88%)

ESBL: extended-spectrum β-lactamase; *: Intermediate and Resistant; **: Combination result of oxacillin screening and penicillin MIC test; MDR: Multi-drug resistant; XDR: Extensively drug resistant; MRSA: Methicillin-resistant Staphylococcus aureus

The total number of isolates submitted in the 2016–2017 period was twice as high as in the 2012–2013 period; for some pathogen-antimicrobial combinations the number of isolates was up to fourfold (*eg.* fourfold for in ESBL, threefold for MRSA). Overall, antimicrobial resistant proportions were higher in 2016–2017 for almost all pathogen-antimicrobial combinations of interest including carbapenem-resistant *A. baumannii*, *P. aeruginosa* and Enterobacterales*.* All Chi-square test returned p-value < 0.0001 that highlight the difference between the two periods; except the combinations with only few isolates (*eg*. comparison of ceftriaxone-resistant *S. pneumoniae*, vancomycin-resistant *E. faecium* and ampicillin-resistant *H. influenzae* proportions from blood and CSF between two periods of VINARES).

**Fig. 2:**
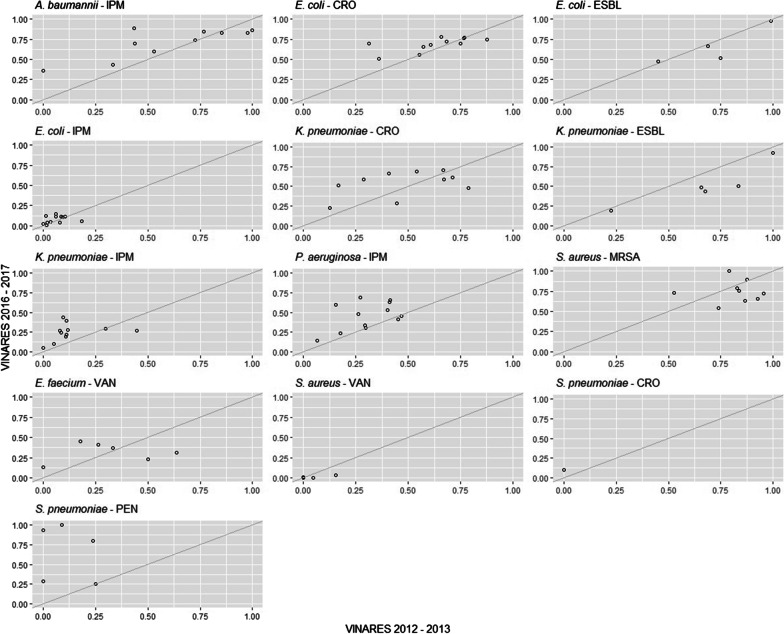
Resistant proportions in 2016–2017 as a function of resistance proportions in 2012–2013 for 13 pathogen-antimicrobial combinations (one per subplot). Each dot corresponds to one of the 13 hospitals that participated in the two VINARES periods. The line is the first diagonal, showing equal proportions of resistance in the 2 periods. IPM: imipenem, CRO: ceftriaxone, VAN: vancomycin, PEN: penicillin

Resistant proportions for 13 pathogen-antimicrobial combinations of 13 hospitals that participated in both periods (2012–13, 2016–17) are shown in Fig. 2. Most hospitals had higher imipenem-resistant *A. baumannii*, *K. pneumoniae, P. aeruginosa* and penicillin non-susceptible *S. pneumoniae* proportions in the second period. ESBL positive Enterobacterales were lower in the second period. No trends for vancomycin-resistant *E. faecium*, ceftriaxone-resistant Enterobacterales and MRSA were seen. The number of AST tests of *A. baumannii*, *K. pneumoniae* and *E. coli* remained unchanged in blood and CSF of 16 or 13 hospitals in VINARES 2012–2013.

The MDR proportion of *E. coli*, *A. baumannii*, *P. aeruginosa* in all specimens were higher in the second period. *K. pneumoniae* MDR went down from 22% (294/1,315 of 13 hospitals) in first period to 14% (428/3,141) in the second. XDR proportions of *E. coli* in all specimens and in blood & CSF were 7% (514/6,956) and 5% (65/1,204), respectively. *K. pneumoniae* had a higher XDR than MDR proportion (23% (722/3,141) in second period compared to 14% of first period of VINARES.

## Discussion

We described the identification and antimicrobial susceptibility testing results from 13 laboratories within the VINARES network in 2016–2017. Overall, we found high proportions of resistance among all tested priority bacteria and these proportions were generally higher than those reported in 2012–2013.

Proportions of carbapenem-resistant Gram-negative pathogens increased gradually in the VINARES hospitals. Carbapenem-resistant *A. baumannii* increased: 40% reported from the GARP report in 2009 [11]; 70% (1,495/2,138) from VINARES 2012–2013 and 79% in the 2016–2017 period. A similar observation can be made for carbapenem-resistant *P. aeruginosa* (30%, 33% and 45%, respectively).

In the 2012 point prevalence survey in 15 hospitals’ ICU in Viet Nam, Phu et al*.* reported that the two most common pathogens of hospital acquired infections (HAI) were *A. baumannii* (24%) and *P. aeruginosa* (14%) [9]. This report showed carbapenem resistance in patients having HAI was most common in *A. baumannii* (89% [149/167]) and *P. aeruginosa* (56% [49/88])[9], similar to our VINARES 2016 data.

In order to understand the situation in Viet Nam from a global perspective, we compared resistant proportions of VINARES 2016–2017 with national AMR surveillance data from LMICs which were submitted to GLASS in 2018 and were published in the GLASS 2020 report [21]. Blood isolates of three countries in Asia (Laos, Cambodia and Myanmar) and two in Africa (Nigeria and Tunisia) were selected for comparison (Additional file 1:  Table 4). Imipenem-resistant *A. baumannii* in blood isolates from Asian countries ranged from 33% (7/21) in Cambodia to 59% (17/29) in Myanmar [21]. In Tunisia, this resistant proportion was 82% (173/210), while data of Nigeria was not available [6]. This proportion was 60% in the VINARES data. The proportions of MRSA remained around 70% in both data periods in VINARES, but was higher than reported from GARP in 2009 (from 17 to 63% in hospitals) [11] and from the Antimicrobial Sensitivity Testing Study in 2006 (42%) [22].

MRSA proportions ranged from 11% (4/35) in Laos to 74% (117/158) in Myanmar [21] in selected Asian LMICs and were 66% (146/222) and 21% (102/483) in Nigeria and Tunisia, respectively [21]. VINARES 2016–2017 had similar MRSA proportions as Myanmar and Nigeria.

Vancomycin-intermediate and resistant *S. aureus* remained stable (2%) over the two time periods of VINARES with no trend observed. Vancomycin-resistance among *S. aureus* was not confirmed molecularly and we are unsure of the significance of these findings.

The decrease in ESBL detection among Enterobacterales was mostly due to changes in use of detection methods. This difference, looking at the denominators for testing between 2012 and 2016, is more likely an artefact of increased ESBL testing using VITEK2 or other automated systems than that they signal a true decrease of ESBL circulation. In 2012–13 ESBL confirmation was only done on a proportion of ceftriaxone resistant isolates in most sites, whereas in 2016–17 a number of sites had switched to using automated systems and almost all isolates were screened for ESBL production.

According to the GLASS 2020 report, ESBL carriage among *E. coli* in Asian and African countries was 30–70%, on par with 59% from VINARES 2016–2017. ESBL carriage among *K. pneumoniae* in VINARES 2016–2017 was 35%, lower in comparison with other countries (38% in Cambodia to 77% in Nigeria) [21].

An increasing trend of penicillin non-susceptible *S. pneumoniae* could not be described properly for the period between 2012–2013 and 2016–2017 period as different methods were used for assessment. There was a change from oxacillin disk diffusion screening in 2012 to penicillin susceptibility test in 2016 across sites. The ANSORP study from 2000 to 2001 reported 91% of penicillin non-susceptible *S. pneumoniae* [23] in Viet Nam, but it may not represent the true prevalence of the entire country because samples were taken in only one hospital in Ho Chi Minh city.

Results from the SOAR study (2009–2011) in 11 centres in Viet Nam reported that 51% (100/195) of *H. influenzae* were resistant to ampicillin [24]. In the VINARES 2016–17 data, ampicillin resistant proportions increased further from 71% in 2012–2013 to 88% in 2016–2017.

Despite the number of hospitals participating in the surveillance network being lower in the second period, the number of isolates submitted was significantly higher. Proportions of AMR were also higher for a number of bug-drug combinations, reflecting the possibly true increases in resistance] over time, the increasing laboratory capacity and the increasing use of microbiology testing as part of diagnostic and antibiotic stewardship programmes. We found a decrease of *K. pneumoniae* MDR in VINARES 2016–2017. This decrease might not reflect an actual trend, but could be explained by the definition of MDR as a *K. pneumoniae* MDR isolate is susceptible to carbapenems by definition and. carbapenem resistance increased.

Our results document a higher proportion of resistance in national than in provincial level hospitals. Previous studies [25–27] have shown that the proportion of patients with hospital-acquired infection is higher in national hospitals. As bacteria associated with hospital acquired infections are usually more resistant, this may partially explain our observation. Furthermore, in accordance with national health recommendations, patients with resistant bacterial infections or patients unresponsive to therapy because of resistance are generally transferred from provincial to national level hospitals, which could further explain the higher levels of resistance in national level hospitals.

### Limitations

VINARES collected isolate-based data (surveillance approaches based solely on laboratory data), without epidemiological, clinical, and population-level data. Currently, GLASS accepts both isolate-based and sample-based data, but it encourages countries to collect and report sample-based data, which can provide stratified and therefore more useful information [6]. Current data collected in VINARES do not allow to differentiate between hospital or community acquired infections. Therefore, resistant proportions may be inflated when trying to use data to inform empiric treatment for community acquired infections. Sample- or case-based data collection may provide potential solutions for this issue.

AST data were collected from isolates cultured from samples sent in for routine diagnostics as part of standard of care. While our study did not make any selection of samples and included all laboratory results, it is known that microbiology is underused in many LMICs for various reasons [28] which may lead to bias, usually towards overestimating resistant proportions.

A standardized sampling and data collection strategy across the whole surveillance network is important to minimize sampling biases, enhance representativeness and interpretation of the results, and allow inference of the results to the country representativeness [6]. The change in the participation of hospitals had impact on the overall resistant proportions.

## Conclusions

We show the results from a successful continuation of a large AMR surveillance network in Viet Nam. The data show alarmingly high and increasing resistant proportions in important organisms causing infections in Viet Nam. However, AMR proportions varied across hospital types in the network. The results may not reflect the true AMR prevalence in Viet Nam as there may be sampling biases and data on whether isolates were from hospital- or community-acquired were not included. Affordable and scalable ways to adopt a sample-or case-based approach across the network should be explored. Clinical data should also be included in the reports from the hospitals to help provide more informative interpretations of the surveillance data.

## Supplementary Information

**Additional file 1.** Supplementary materials.

## Data Availability

The data sharing agreements are in place for aggregated data with ResistanceMap (www.resistancemap.org) and in preparation for individual level data with the the Global Burden of Disease / GRAM project on AMR. We have no agreement from the hospitals to make individual level data publicly available.
