# Supplementary material for: Antimicrobial susceptibility testing results from 13 hospitals in Viet Nam: VINARES 2016–2017
Source: Antimicrob Resist Infect Control. 2021 May 10;10:78. doi: 10.1186/s13756-021-00937-4 (PMC8112055; doi:10.1186/s13756-021-00937-4)
Supplement: Supplementary file 1 — Additional file 1. Supplementary materials. [file 13756_2021_937_MOESM1_ESM.docx]

# Supplementary materials

Supplementary table 1: Proportions of MIC among AST tests done by disk diffusion and MIC methods in the 13 laboratories that participated in the two periods. Denominators are the numbers of AST tests by both disk diffusion and MIC methods, numerators are the numbers of MIC tests. Corresponding MIC proportions are in bracket. MRSA were combined from cefoxitin and oxacilin; penicillin susceptibility testing was combined from penicillin and oxacillin results. Laboratories were numbered from 1 to 13 (these number are not the same as in figure 1 – as we did not show results of individual laboratory) for those participating in the two periods.

|  | MRSA | | *E. coli* - IPM | | *K. pneumoniae* - IPM | | *A. baumannii* - IPM | | *P. aeruginosa* - IPM | | *A. baumannii* - CAZ | | *S. pneumoniae* PEN | | |
| --- | --- | --- | --- | --- | --- | --- | --- | --- | --- | --- | --- | --- | --- | --- | --- |
| Lab | 2012-2013 | 2016-2017 | 2012-2013 | 2016-2017 | 2012-2013 | 2016-2017 | 2012-2013 | 2016-2017 | 2012-2013 | 2016-2017 | 2012-2013 | 2016-2017 | 2012-2013 | 2016-2017 |  |
| 01 | 0/583  (0) | 0/396 (0) | 0/344  (0) | 0/687 (0) | 0/268  (0) | 0/251 (0) | 0/211  (0) | 0/17 (0) | 0/108  (0) | 0/234 (0) | 0/168  (0) | 0/19 (0) | 0/5  (0) | 0/18 (0) |  |
| 02 | 0/650  (0) | 0/424 (0) | 0/488  (0) | 0/677 (0) | 0/285  (0) | 0/46 (0) | 0/318  (0) | 0/46 (0) | 0/184  (0) | 0/262 (0) | 0/318  (0) | 0/46 (0) | 0/44  (0) | 0/2 (0) |  |
| 03 | 0/115  (0) | 943/943 (100) | 0/81  (0) | 3042/3042 (100) | 0/99  (0) | 1016/1016 (100) | 0/350  (0) | 1796/1796 (100) | 0/47  (0) | 557/557 (100) | 0/351  (0) | 1796/1796 (100) | 0/0 | 14/15 (93) |  |
| 04 | 0/541  (0) | 0/103 (0) | 0/84  (0) | 0/297 (0) | 0/101  (0) | 0/295 (0) | 0/0  -- | 0/303 (0) | 0/0 | 0/151 (0) | 0/0 | 2/2 (100) | 0/5  (0) | 0/1 (0) |  |
| 05 | 0/34  (0) | 0/337 (0) | 0/72  (0) | 0/360 (0) | 0/37  (0) | 0/258 (0) | 0/17  (0) | 0/57 (0) | 0/26  (0) | 0/187 (0) | 0/17  (0) | 0/55 (0) | 0/3  (0) | 0/26 (0) |  |
| 06 | 0/1099  (0) | 0/68 (0) | 0/218  (0) | 0/189 (0) | 0/166  (0) | 0/129 (0) | 0/132  (0) | 0/170 (0) | 0/82  (0) | 0/102 (0) | 0/230  (0) | 0/164 (0) | 1/2  (50) | 0/0 |  |
| 07 | 213/277  (77) | 0/372 (0) | 113/135  (84) | 0/915 (0) | 27/38  (71) | 0/255 (0) | 27/38  (71) | 0/134 (0) | 38/53  (72) | 0/196 (0) | 27/38  (71) | 0/132 (0) | 7/11  (64) | 0/70 (0) |  |
| 08 | 0/411  (0) | 0/394 (0) | 0/173  (0) | 0/387 (0) | 0/108  (0) | 0/395 (0) | 0/103  (0) | 0/263 (0) | 0/65  (0) | 0/218 (0) | 0/101  (0) | 0/294 (0) | 0/57  (0) | 50/56 (89) |  |
| 09 | 0/105  (0) | 0/123 (0) | 0/129  (0) | 0/409 (0) | 0/63  (0) | 0/98 (0) | 0/46  (0) | 0/4 (0) | 0/44  (0) | 0/106 (0) | 0/31  (0) | 0/4 (0) | 0/2  (0) | 0/0 |  |
| 10 | 0/84  (0) | 0/164 (0) | 0/99  (0) | 0/172 (0) | 0/134  (0) | 0/324 (0) | 0/161  (0) | 0/387 (0) | 0/143  (0) | 1/211 (0) | 0/159  (0) | 3/387 (1) | 0/1  (0) | 35/35 (100) |  |
| 11 | 0/265  (0) | 0/334 (0) | 0/0 | 0/535 (0) | 0/154  (0) | 0/313 (0) | 0/37  (0) | 0/251 (0) | 0/101  (0) | 0/431 (0) | 0/37  (0) | 0/261 (0) | 0/34  (0) | 54/237 (23) |  |
| 12 | 0/329  (0) | 111/612 (18) | 0/56  (0) | 378/476 (79) | 0/16  (0) | 224/256 (88) | 0/1  (0) | 100/112 (89) | 0/31  (0) | 120/200 (60) | 0/11  (0) | 114/124 (92) | 75/173  (43) | 540/643 (84) |  |
| 13 | 0/239  (0) | 0/245 (0) | 0/232  (0) | 0/292 (0) | 0/228  (0) | 0/11 (0) | 0/170  (0) | 0/11 (0) | 0/112  (0) | 0/365 (0) | 0/195  (0) | 0/14 (0) | 0/4  (0) | 1/33 (3) |  |
|  |  |  |  |  |  |  |  |  |  |  |  |  |  |  |  |

MIC: Minimum inhibitory concentration; AST: Antimicrobial susceptibility testing; MRSA: methicillin-resistant Staphylococcus aureus; IPM: Imipenem; CAZ: ceftazidime; PEN: penicillin.

Table 2a: Antimicrobial susceptibility testing results of three gram-positive bacteria isolated from ICU specimens of 13 hospitals in VINARES 2016-2017 project. Denominators and numerators are the numbers of tested and of resistant isolates respectively. Corresponding resistant percentages are in brackets.

|  | *S. aureus* (N=690) | *S. pneumoniae* (N=184) | *E. faecium* (N=65) |
| --- | --- | --- | --- |
| Aminoglycosides | 250/468 (53%) |  | 6/6 (100%) |
| Fluoroquinolones | 331/647 (51%) | 2/137 (1%) |  |
| Macrolides | 548/660 (83%) | 154/167 (92%) | 57/59 (97%) |
| MRSA | 478/640 (75%) |  |  |
| Penicillin | 313/324 (97%) | 42/146 (29%) | 20/24 (83%) |
| SXT | 116/615 (19%) | 103/130 (79%) | 2/2 (100%) |
| Ampicillin |  |  | 50/54 (93%) |
| Azithromycin | 101/123 (82%) | 7/10 (70%) |  |
| Vancomycin* | 17/340 (5%) | 1/146 (1%) | 22/64 (35%) |

MRSA: Methicillin-resistant Staphylococcus aureus; SXT: Trimethoprim/Sulfamethoxazole

Table 2b: Antimicrobial susceptibility testing results of eight gram-negative bacteria isolated from ICU specimens of 13 hospitals in VINARES 2016-2017 project. Denominators and numerators are the number of tested and resistant isolates respectively. Corresponding resistant percentages are in brackets.

|  | *A. baumannii* (N=1176) | *P. aeruginosa* (N=1158) | *K. pneumoniae* (N=1069) | *E. coli* (N=1016) | *Enterobacter spp.* (N=230) | *H. influenzae* (N=146) | *Salmonella spp.* (N=18) | *Shigella spp.* (N=10) |
| --- | --- | --- | --- | --- | --- | --- | --- | --- |
| Carbapenem | 976/1132 (86%) | 664/1128 (59%) | 415/1051 (39%) | 146/1004 (15%) | 96/229 (42%) | 0/146 (0%) | 0/12 (0%) | 0/1 (0%) |
| Aminoglycosides | 962/1135 (85%) | 604/1136 (53%) | 632/1032 (61%) | 422/998 (42%) | 128/229 (56%) |  | 2/5 (40%) | 1/1 (100%) |
| Fluoroquinolones | 958/1103 (87%) | 597/1126 (53%) | 570/973 (59%) | 568/950 (60%) | 120/224 (54%) | 6/117 (5%) | 2/17 (12%) | 2/7 (29%) |
| Cephalosporins | 992/1076 (92%) | 554/1014 (55%) | 726/1033 (70%) | 579/925 (63%) | 139/201 (69%) | 2/48 (4%) | 1/16 (6%) | 3/4 (75%) |
| Macrolides | 17/18 (94%) |  |  |  |  | 3/134 (2%) | 1/6 (17%) | 2/3 (67%) |
| ESBL |  |  | 557/1035 (54%%) | 603/996 (61%%) | 161/218 (74%%) |  |  |  |
| SXT |  | 332/351 (95%) | 629/937 (67%) | 700/925 (76%) | 121/204 (59%) | 111/120 (92%) | 5/18 (28%) | 10/10 (100%) |
| AMC |  |  | 527/781 (67%) | 259/489 (53%) | 52/58 (90%) | 64/94 (68%) |  |  |
| Ampicillin |  |  | 696/704 (99%) | 556/584 (95%) | 123/126 (98%) | 92/98 (94%) | 10/17 (59%) | 10/10 (100%) |
| TCC |  | 436/693 (63%) | 491/696 (71%) | 313/646 (48%) | 85/158 (54%) |  |  |  |
| MDR | 895/1018 (88%) | 270/461 (59%) | 143/790 (18%) | 158/736 (21%) |  |  |  |  |
| XDR |  |  | 295/790 (37%) | 61/736 (8%) |  |  |  |  |

ESBL: extended-spectrum β-lactamase; SXT: Trimethoprim/Sulfamethoxazole; AMC: amoxicillin clavulanic acid; TCC: Ticarcillin/Clavulanic Acid; *: Resistant and Intermediate; MDR: Multi-drug resistant; XDR: Extensively drug resistant.

Table 3: Antimicrobial susceptibility testing results by hospital type. Denominators and numerators are the numbers of tested resistant isolates, respectively. Corresponding resistant percentages are in brackets.

|  | *E. coli* (N=4001) | | | *Acinetobacter spp.* (N=2469) | | | *S. aureus* (N=1534) | | |
| --- | --- | --- | --- | --- | --- | --- | --- | --- | --- |
|  | National | Provincial | Specialised | National | Provincial | Specialised | National | Provincial | Specialised |
| Carbapenem | 466/3801 (12%) | 370/3253 (11%) | 125/1776 (7%) | 1979/2413 (82%) | 432/632 (68%) | 444/577 (77%) |  |  |  |
| Cephalosporins | 2670/3541 (75%) | 1730/2884 (60%) | 1041/1770 (59%) | 2053/2377 (86%) | 464/637 (73%) | 452/535 (84%) |  |  |  |
| ESBL | 2145/3726 (58%) | 1541/2371 (65%) | 399/856 (47%) |  |  |  |  |  |  |
| MRSA |  |  |  |  |  |  | 1115/1440 (77%) | 1499/2115 (71%) | 688/960 (72%) |

ESBL: extended-spectrum β-lactamase; MRSA: Methicillin-resistant Staphylococcus aureus.

Table 4: Comparison MRSA, ESBL and CRE proportion of LMIC (data submitted in 2018, published in GLASS 2020 report [6]) and Viet Nam (VINARES 2016-2017) in blood isolates. For other countries apart from Viet Nam, MRSA and ESBL were determined using cefoxitin non-susceptible and ceftazidime resistant, respectively.

|  | MRSA | ESBL *E.coli* | ESBL *K. pneumoniae* | Imipenem-resistant *E. coli* | Imipenem-resistant  *A. baumannii* |
| --- | --- | --- | --- | --- | --- |
| Laos | 4/35 (11%) | 29/65 (45%) | 10/13 (77%) | NA | NA |
| Cambodia | 20/41 (49%) | 109/253 (43%) | 36/94 (38%) | 11/210 (5%) | 7/21 (33%) |
| Myanmar | 117/158 (74%) | 15/20 (75%) | NA | 9/52 (17%) | 17/29 (59%) |
| Nigeria | 146/222 (66%) | 31/44 (70%) | 44/57 (77%) | NA | NA |
| Tunisia | 102/483 (21%) | 59/194 (30%) | 200/300 (67%) | 1/248 (0.4%) | 173/210 (82%) |
| Viet Nam  (VINARES 2016-2017) * | 476/674 (71%) | 655/1107 (59%) | 128/365 (35%) | 116/1483 (8%)** | 110/183 (60%)** |

ESBL: extended-spectrum β-lactamase; MRSA: Methicillin-resistant Staphylococcus aureus; NA: Not available; *: blood and CSF; **: Imipenem or imipenem- or meropenem- or ertapenem- resistant.
